# Effect of Select Tannin Sources on Pathogen Control and Microbial Nitrogen Metabolism in Composted Poultry Litter Intended for Use as a Ruminant Crude Protein Feedstuff

**DOI:** 10.3389/fvets.2022.930980

**Published:** 2022-06-21

**Authors:** Claudio Arzola-Alvarez, Robin C. Anderson, Michael E. Hume, Evelyn Ledezma, Oscar Ruiz-Barrera, Yamicela Castillo-Castillo, Alejandro Arzola-Rubio, Marina Ontiveros-Magadan, Byeng Ryel Min, Lauren R. Wottlin, Ramon Copado, Jamie Salinas-Chavira

**Affiliations:** ^1^College of Animal Science and Ecology, Autonomous University of Chihuahua, Chihuahua, Mexico; ^2^United States Department of Agriculture, Agricultural Research Service, Food and Feed Safety Research Unit, Southern Plains Agricultural Research Center, College Station, TX, United States; ^3^Agricultural and Environmental Sciences, Tuskegee University, Tuskegee, AL, United States; ^4^College of Veterinary Medicine, Autonomous University of Nuevo Leon, Monterrey Nuevo Leon, Mexico; ^5^College of Veterinary Medicine, Universidad Autónoma de Tamaulipas, Matamoros, Mexico

**Keywords:** *Escherichia coli*, nitrogen metabolism, poultry litter, *Salmonella*, tannins

## Abstract

Poultry litter is a good crude protein supplement for ruminants but must be treated to kill pathogens before feeding. Composting effectively kills pathogens but risks loss of ammonia due to uric acid degradation. The objectives of this study were to test the ability of tannins to reduce pathogens and preserve uric acid during poultry litter composting. In two experiments, poultry litter was mixed with phosphate buffer and distributed to 50-ml tubes (three tubes/treatment per sample day) amended with 1 ml buffer alone or buffer containing pine bark, quebracho, chestnut, or mimosa tannins. Treatments achieved 0.63% (wt/wt) quebracho, chestnut, or mimosa tannins in experiment 1, or 4.5% pine bark or 9% quebracho, chestnut, or mimosa tannins in experiment 2. Tubes were inoculated with a novobiocin- and nalidixic acid-resistant *Salmonella* typhimurium, closed with caps, and incubated at successive 3-day increments at 22, 37, and 42°C, respectively. In experiment 1, bacterial counts in contents collected on days 0, 6, and 9 revealed a treatment by day effect (*p* < 0.03), with the *Salmonella* challenge being 1.3 log_10_ CFU/g higher in quebracho-treated composts than in untreated controls after 6 days of composting. After 9 days of composting, *Salmonella*, wildtype *Escherichia coli*, and total aerobes in untreated and all tannin-treated composts were decreased by about 2.0 log_10_ CFU/g compared to day 0 numbers (3.06, 3.75, and 7.77 log_10_ CFU/g, respectively). Urea and ammonia concentrations tended (*p* < 0.10) to be increased in chestnut-treated composts compared to controls and concentrations of uric acid, urea, and ammonia were higher (*p* < 0.05) after 9 days of composting than on day 0. Despite higher tannin application in experiment 2, antibacterial effects of treatment or day of composting were not observed (*p* > 0.05). However, treatment by time of composting interactions was observed (*p* < 0.05), with quebracho- and chestnut-treated composts accumulating more uric acid after 24 h and 9 days of composting and chestnut-, mimosa- or quebracho-treated composts accumulating less ammonia than untreated composts. Results demonstrate that composting may effectively control pathogens and that tannin treatment can help preserve the crude protein quality of composting poultry litter.

## Introduction

The continuing increase in poultry production brings challenges for producers to implement appropriate environmentally sustainable methods to dispose of the huge amounts of wastes accompanying intensive production practices (1, 2). Broiler and egg production yield huge amounts of poultry excreta often mixed with bedding material, feathers, insects, uneaten feed and, in the case of laying hens, the shells and contents of broken eggs (3). The high concentration of nitrogen in the litter, which is present largely in the form of uric acid, makes it a valuable fertilizer and antimicrobial-resistant microbes within the litter necessitates that the material must be treated before ruminant crude protein feedstuff, although the presence of dangerous pathogens feeding (4). In the United States, the feeding of poultry litter has lost favor among many cattle producers due largely to concerns that adverse consumer perceptions associated with feeding litter to animals may lower consumer demand for beef products. However, a contrasting approach to this view may be applied as the innovative development of environmentally safe and sustainable technologies to conserve limited resources by recycling nutrients contained in poultry litter is arguably consistent not only with good agricultural practices but with green technologies intended to support increased animal food production for our growing world population.

Bakshi and Fontenot (5) were early to report systematic studies on the effective elimination of pathogens with the composting of poultry litter, achieved through increases in temperature and lactic acid, and the lowering of the pH during the composting process. Composting, however, can result in appreciable losses in nitrogen via the breakdown of uric acid, urea, and other fixed nitrogen sources to yield ammonia, which, depending on pH, can be volatilized into the atmosphere or it can leach away into the soil (5–7). Consequently, technologies are sought to preserve the crude protein value within poultry litter during composting. Recent research reported that chemical treatment of poultry litter with certain nitrocompounds decreased ammonia losses by 17 to 24% and increased uric acid accumulations by 18% during 9 days of composting (8). Additionally, the concentration of urea in litter treated with 3-nitropropionate, a naturally occurring compound found in certain leguminous forages as well as certain fungi, was increased by nearly 50% from initial concentrations after the 9-day compost period (8). Similarly, Steiner et al. (9) demonstrated that the addition of commercial pine chip biochar to composting broiler litter reduced total nitrogen losses by almost 25%. More recently, lignite addition to poultry litter increased total nitrogen by 25% and achieved >60% increase in mineral nitrogen (10). Arzola-Alvarez et al. (11) reported that the inclusion of pine bark tannin in composting poultry litter decreased ammonia losses by 23% and increased uric acid concentrations by 63% during 3 days of composting. The pine bark tannin and some of the nitrocompound treatments also accelerated anti-*Salmonella* activity, achieving significant, albeit modest, decreases in *Salmonella* counts by 3 days of composting when compared to controls, although this may be of limited benefit as effectively managed composts usually eliminate *Salmonella* after 14 days of composting if not earlier (12, 13). Considering that tannins are natural sources recognized for their ability to mitigate nitrogen metabolism in anaerobic environments, the objectives of the present experiments were to assess the antimicrobial and nitrogen retaining effects of condensed or hydrolyzed tannin-rich extracts common to ruminant diets when applied to simulated litter composts.

## Materials and Methods

### Rearing, Source of Litter, and *Salmonella* typhimurium Inoculum

Used wood chip poultry litter was graciously provided by the Texas A&M University poultry facility. The litter used in the first experiment was 1-year-old and was used to rear seven broiler flocks. The litter used in the second experiment was approximately 4 months old and had been used to rear two broiler flocks. Litter in both cases had been used without antibiotic or coccidiostat exposure and was screened through a 17-mm diameter sieve to omit oversized particles before use. The dry matter content, determined after 24 h in a 100°C oven, was 76 and 98% for litter used in the first and second experiments, respectively.

Pine bark tannin (containing about 10% condensed tannin) was obtained via acetone extraction from pine bark and subsequent fractionation using Sephadex LH-20 (14). Chestnut (containing about 80% hydrolyzable tannins), mimosa and quebracho tannins (containing about 70 and 75% condensed tannins, respectively) were each procured from Chemtan Company, Inc. (Exeter, NH, USA). The challenge *Salmonella enterica* serovar typhimurium strain (NVSL 95-1776), possessing natural resistance to novobiocin, had been made nalidixic acid-resistant via successive cultivation in broth containing up to 20 μg nalidixic acid/ml (15). The inocula for each of the two experiments in this study were obtained from cultures grown overnight at 37°C in tryptic soy broth (Difco, Becton Dickinson, Sparks, MD, USA) supplemented with 25 and 20 μg/ml of novobiocin and nalidixic acid, respectively. Both antibiotics were purchased from Sigma-Aldrich (St. Louis, MO, USA).

### Experimental Design of Simulated Poultry Litter Composts

In the first experiment, freshly collected broiler litter was weighed (200 g/tray) and spread to equally cover the bottoms of five separate 26 cm × 39 cm × 8 cm plastic trays. Each tray was then sprayed with 80 ml of 0.4 M phosphate buffer (pH 6.5) alone or phosphate buffer containing 0.95 g of pine bark, quebracho, chestnut, or mimosa tannin using separate hand spray bottles to achieve effective coverage of 0.63% tannin (wt/wt) of litter dry matter. The sprayed litter preparations were each mixed by hand and then distributed (11 g) to 50-ml tubes (nine tubes/treatment to achieve three tubes/treatment per sample day) and amended with 1.1 ml of phosphate buffer containing 10^5^ colony forming units (CFU)/ml of the novobiocin- and nalidixic acid-resistant *S*. typhimurium. The tubes were closed with caps and incubated at successive 3-day increments at 22, 37, and 42°C, respectively, to simulate conditions commonly encountered during composting. For all incubations, the tubes were placed in BBL Anaerobic Gas Pack (Becton Dickinson, Sparks, MD, USA) containers and incubated aerobically from days 0 to 3 then flushed with 100% with CO_2_ and incubated under this atmosphere for days 3 to 9. The incubation atmosphere protocol was designed to simulate conditions commonly encountered during composting.

In the second experiment, 50 to 100 g of freshly collected broiler litter was spread to equally cover the bottoms of plastic trays as described above and then sprayed with 20 or 40 ml of 0.4 M phosphate buffer (pH 6.5) alone (controls) or phosphate buffer containing 4.5 g of pine bark or 9.0 g of quebracho, chestnut or mimosa tannins to achieve treatment levels of 4.5 or 9% tannin (wt/wt) of litter dry matter. In this case, the sprayed litter preparations were each mixed by hand and then distributed (5 g) to 50-ml tubes (nine tubes/treatment to achieve three tubes/treatment per sample day) and amended with 1.1 ml of phosphate buffer containing 10^5^ CFU/ml of the novobiocin- and nalidixic acid-resistant *S*. typhimurium. Smaller amounts of litter were used in the second experiment to accommodate administering higher treatment amounts with limited availability of the tannin sources and in particular the availability of pine bark tannin which was sufficient only to achieve an application amount (4.5% of litter dry matter) whereas an application amount of 9% of litter dry matter was achieved with the other tannin sources. The tubes were closed with caps and incubated as described above. Tubes were collected for sampling at 0 time and, in anticipation of potentially more potent and immediate activation of the higher tannin amounts than used in the first experiment, after 0.25, 1, and 9 days of composting (the equivalent of 0, 6, 24, and 216 h, respectively). Litter in collected tubes was processed immediately for the quantitative determination of the challenge *S*. typhimurium, the wildtype *E. coli* population, and total aerobes. Fluid samples (1.0 ml) were also obtained at the time of tube collection and frozen at −20°C for subsequent determination of uric acid, urea, and ammonia concentrations.

### Analytical Methods

Tubes collected after 0, 6, and 9 days of composting in the first experiment and after 0, 0.25, 1, and 9 days of composting in the second experiment were initially amended with 22 or 10 ml 0.4 M phosphate buffer (pH 6.5), respectively, at time of collection and vortexed for 1 min to dislodge cell-associated microbes. About 500 μl of the initial dilution and 100 μl of subsequent 10-fold dilutions (out to 10^−5^ for the *S*. typhimurium challenge strain and out to 10^−6^ and 10^−7^ for the wildtype *E. coli* population and total aerobes, respectively) were plated within 30 min of tube collection to respective media for bacterial enumeration. For the challenge *S*. typhimurium, which was phenotypically resistant to novobiocin and nalidixic acid, dilutions were plated on brilliant green agar (Oxoid Ltd., Basingstoke, Hampshire, England) containing, respectively, 20 and 25 μg/ml of novobiocin and nalidixic acid. Wildtype *E. coli* and total culturable aerobes were enumerated via plating serial dilutions on 3 M *E. coli*/coliform petrifilm and 3 M aerobic plate count petrifilm, respectively (3 M, Minneapolis, MN, USA), the latter not discriminating between obligate or facultative aerobes. Plates were incubated and recorded according to manufacturers' instructions.

The 1-ml aliquots of fluid collected above and frozen were subjected to colorimetric determination of concentrations of uric acid using the Uric Acid Assay kit (Sigma-Aldrich), for urea using the Quantichrom^TM^ Urea Assay Kit (BioAssay Systems, Hayward, CA, USA), and for ammonia as described by Chaney and Marbach (16). Because of the higher amounts of tannins added to the composts in the second experiment, 200-μl aliquots of collected fluid samples were mixed and reacted for 30 min with 1 g 100% ethanol-swelled Sephadex LH-20 (cytiva, Global life sciences IP Holdco LLC, Upsala, Sweden) to remove residual tannin prior to colorimetric analysis.

### Denaturing Gradient Gel Electrophoresis (DGGE)

To assess microbial band patterns as influenced by treatments in the first experiment, genomic DNA was extracted from the tubes of control composts collected at 0 time (representative of pre-treatment populations) and from tubes of controls and treated composts at the 6 and 9 days collection periods. Extraction of DNA was accomplished according to the protocol of the NucleoSpinVR Tissue kit (Macherey-Nagel, Germany) and was quantified using an ENDUROVR Touch Gel Documentation system, Labnet, Edison NJ. Polymerase chain reaction (PCR) amplification of the V3 region of the 16S rRNA gene for DGGE analysis of the bacterial band patterns was carried out according to the methods described by Muyzer and Smalla (17) and Hume et al. (18). Differences in bacterial populations were determined by analysis of band patterns. Diversity comparisons are presented descriptively by showing dice percentage similarity coefficients (%SC) and dendrograms constructed by an unweighted pair group method using the arithmetic averages (UPGMA) options in Gel Compare II 6.6 (Applied Maths, Inc., Austin, TX, USA).

### Statistical Analysis

Each compost tube, which was prepared, incubated, and sampled independent of the other compost tubes, served as an independent experimental unit. Bacterial concentrations of litter were log_10_-transformed to normalize variances. A general analysis of variance was used to test the main effects of treatment, sample day, and their potential interaction on log_10_ transformations of bacterial CFU/g of litter and on concentrations (μmol/g litter) of uric acid, urea, and ammonia using STATISTIX 10^TM^ (Tallahassee, FL, USA). Comparisons of means were accomplished using an LSD all-pairwise comparison test. Significance was declared at *p* ≤ 0.05, and statistical tendencies were declared at 0.05 > *p* ≤ 0.10.

## Results

### Tannin Effects on Select Microbial Populations in Composted Broiler Litter

**Figure 1:**
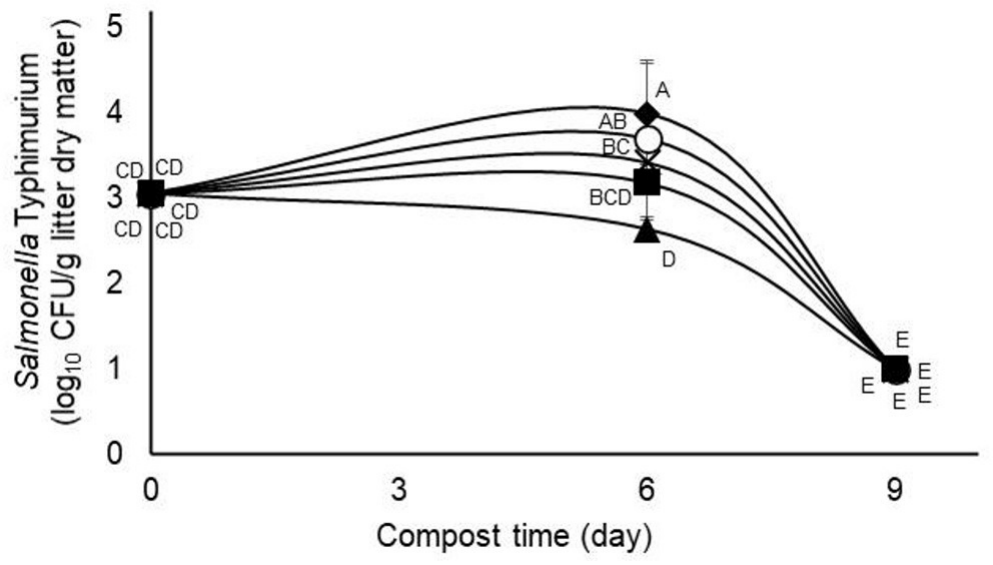
Interaction (*p* = 0.0156) between treatment and day of composting on the challenge *Salmonella* typhimurium during 9-days simulated composting of poultry litter treated without (open circles) or with (0.63%) pine bark- (closed squares), quebracho- (closed triangles), chestnut- (closed diamonds), or mimosa- (X) tannins (Experiment 1). Means with unlike uppercase letters differ based on an LSD all pairwise test at *p* < 0.05. Error bars denote standard deviations from *n* = 3 independent replicates at each time point.

**Figure 2:**
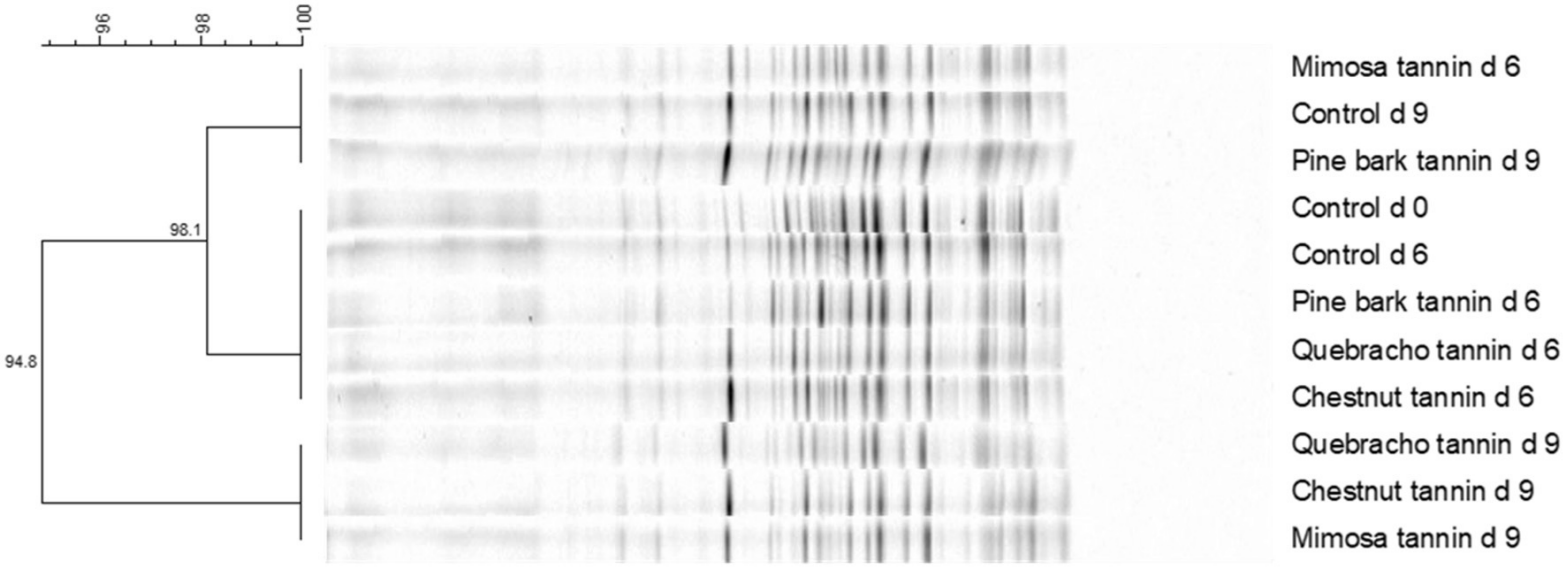
Denaturing gradient gel electrophoresis dendrogram showing effects of 0.63% tannin treatment on microbial diversity (Experiment 1).

The main effect of administering tannin treatments at 0.63% of litter dry matter during the first composting experiment was observed on recovery of the challenge *S*. typhimurium strain but not on numbers of *E. coli* or total aerobes (Table 1). In the case of the challenge *S*. typhimurium, counts were lowest (*p* < 0.05) in the quebracho-treated composts, highest (*p* < 0.05) in the chestnut-treated and untreated composts, and intermediate in the pine bark- and mimosa-treated composts (Table 1). Conversely, the main effect of time of composting was observed (*p* < 0.05) on the numbers of all measured microbial populations, with the challenge *S*. typhimurium and *E. coli* decreasing to below detectable levels regardless of treatment after 9 days of composting when compared to counts measured on day 0 (Table 2). Populations of total culturable aerobes decreased more than 1.8 log_10_ CFU/g of litter dry matter (Table 2). Numbers of *E. coli* were lower (*p* < 0.05) on day 6 of composting when compared to day 0 but numbers of the challenge *S*. typhimurium and total aerobes were either increased (*p* < 0.05) or not significantly different (*p* > 0.05), respectively, on day 6 when compared to day 0 (Table 2). A treatment by time of composting interaction was observed on recovery of the challenge *S*. typhimurium, with day 6 counts being lower than untreated controls only in the compost treated with 0.63% quebracho (Figure 1). Treatment by the time of composting interactions was not observed on numbers of *E. coli* or total culturable aerobes (not shown). Tannin treatment and day of composting had only modest effects on community microbial diversity within the composted poultry litter, with populations among controls and all treatments being more than 94% similar based on calculated similarity coefficients (Figure 2).

**Table 1:** Main effect treatment means tannin treatment on bacterial counts and accumulations of uric acid, urea, and ammonia during a 9-day simulated compost of wood chip broiler litter (Experiment 1).

	**Bacterial counts**				**Nitrogen concentrations**		
	**(log**_**10**_ **CFU/g of litter dry matter)**				**(μmol/g litter dry matter)**		
**Tannin treatment (% Litter dry matter)**	**Challenge *Salmonella* typhimurium**	**Wildtype *Escherichia coli***	**Wildtype total culturable aerobes**		**Uric acid**	**Urea**	**Ammonia**
None	2.59^a^	2.66	7.22		2.70	1.52	1.62
0.63% pine bark	2.41^ab^	2.54	7.22		2.34	1.90	1.62
0.63% quebracho	2.23^b^	2.44	7.04		2.24	1.62	1.52
0.63% chestnut	2.68^a^	2.48	7.13		2.75	2.18	1.74
0.63% mimosa	2.49^ab^	2.50	6.96		2.63	1.80	1.62
Treatment effect (*p*-value)	0.0367	0.7852	0.3678		0.5332	0.0549	0.0552
SEM^c^	0.1076	0.1284	0.1091		0.2527	0.1591	0.0492

^a, b^*Means (n = 3) within columns with unlike superscripts differ significantly, p <0.05; ^c^SEM, standard error of the mean from independent replicates*.

**Table 2:** Main effect means of day of composting on bacterial counts and accumulations of uric acid, urea, and ammonia during a 9-day simulated compost of wood chip broiler litter treated without or with 0.63% pine bark, quebracho, chestnut, or mimosa tannins per g litter dry matter (Experiment 1).

	**Bacterial counts**				**Nitrogen concentrations**		
	**(log**_**10**_ **CFU/g of litter dry matter)**				**(μmol/g litter dry matter)**		
**Days of composting**	***Salmonella* typhimurium**	**Wildtype *Escherichia coli***	**Wildtype total Culturable aerobes**		**Uric acid**	**Urea**	**Ammonia**
0	3.06^b^	3.75^a^	7.77^a^		1.98^b^	1.50^b^	1.34^c^
6	3.38^a^	2.82^b^	7.70^a^		2.88^a^	1.70^b^	1.71^b^
9	1.00^c^	1.00^c^	5.88^b^		2.73^a^	2.31^a^	1.82^a^
Treatment effect (*p*-value)	<0.0001	<0.0001	<0.0001		0.0064	0.0009	<0.0001
SEM^d^	0.0809	0.0995	0.0845		0.2527	0.1591	0.0492

^a, b, c^*Means (n = 3) within columns with unlike superscripts differ significantly, p <0.05; ^d^SEM, standard error of the mean from independent replicates*.

**Figure 3:**
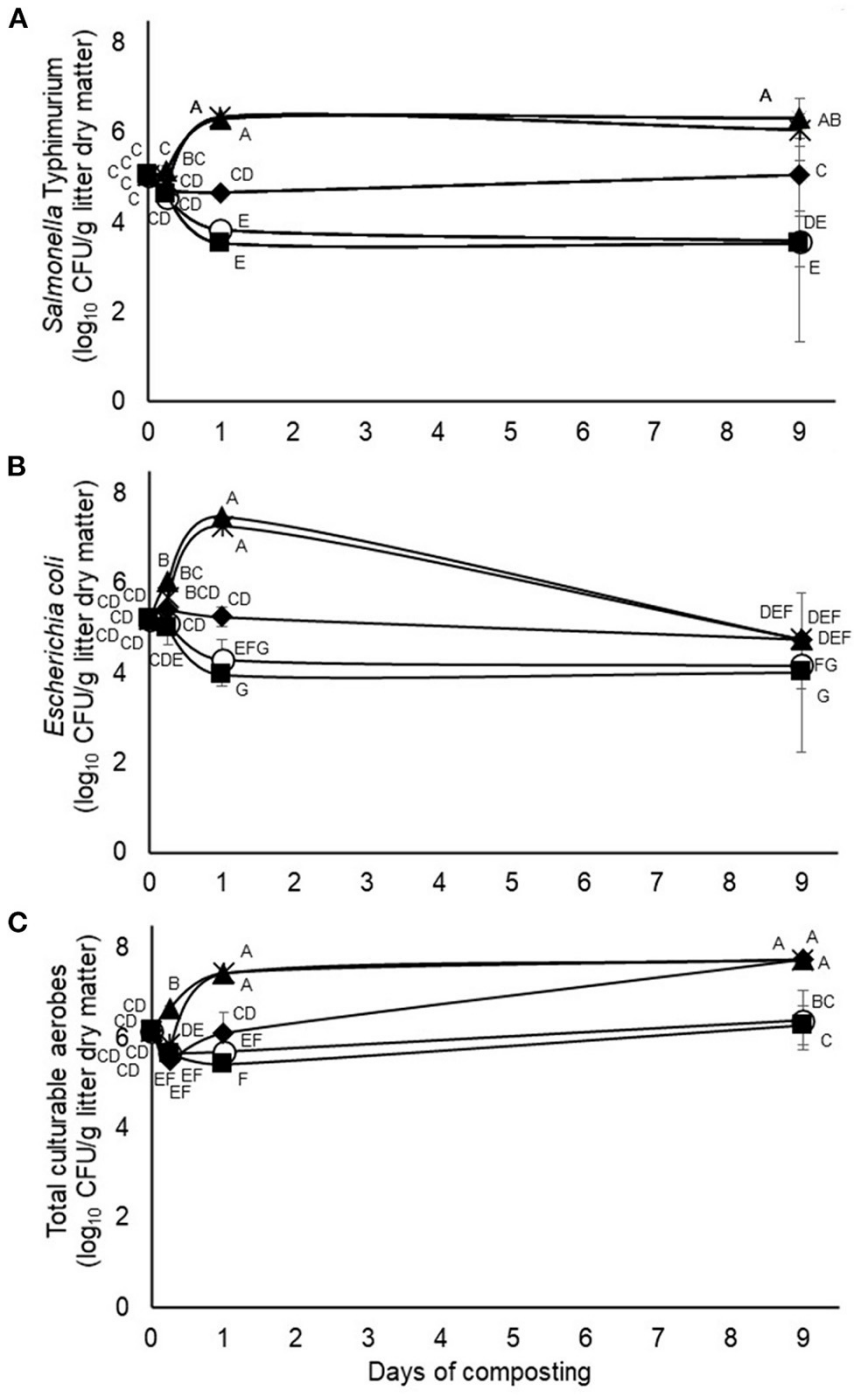
Interactions (*p* = 0.0001, <0.0001, and <0.0001, respectively) between treatment and time of composting on counts of the challenge *Salmonella* typhimurium **(A)**, wildtype *E. coli* **(B)** and total culturable aerobes **(C)** during 9-days simulated composting without (open circles) or with 4.5% pine bark (closed squares) or 9% quebracho (closed triangles), chestnut (closed diamonds) or mimosa (X) tannins (Experiment 2). Means with unlike uppercase letters differ based on an LSD all pairwise comparison test at *p* < 0.05. Error bars denote standard deviations from *n* = 3 independent replicates at each time point.

Results from the second experiment, testing application of 4.5% of pine bark tannin and 9% of quebracho, chestnut, or mimosa tannins, respectively, revealed treatment by time of composting interactions on viable numbers of the challenge *S*. typhimurium as well as on the wildtype *E. coli* and total culturable aerobe populations (Figure 3). In this case, however, all three of the measured microbial populations qualitatively appeared to be much more resilient to composting than in the first experiment despite the application of much higher tannin doses in the second experiment (4.5 to 9% added tannin vs. 0.63% added tannin, respectively). Moreover, composts treated with quebracho or mimosa tannins exhibited an increase (*p* < 0.05) in numbers of the different microbial populations, with numbers increasing as early as 6 to 24 h of composting and persisting at higher (*p* < 0.05) numbers than the untreated composts even after 9 days, although less dramatically for *E. coli* than the challenge *S*. typhimurium or total aerobes (Figure 3). Conversely, composts treated with pine bark tannin exhibited little (*p* > 0.05) if any effect on numbers of the measured microbial populations whereas composts treated with chestnut tannins were intermediate, being slower to enrich the microbial populations than composts treated with quebracho or mimosa tannins (Figure 3).

### Tannin Effect Upon Nitrogen in Composted Broiler Litter

The main effect of treatment or treatment by time of composting interactions was not observed (*p* > 0.05) on nitrogen accumulations in the first composting experiment testing 0.63% tannin administration, although concentrations of urea and ammonia tended to be higher (0.05 < *p* < 0.10) in chestnut-treated than untreated composts (Table 1). The main effect of time of composting was observed on nitrogen accumulations, with concentrations of uric acid, urea, and ammonia being increased (*p* < 0.05) after 6 and 9 days of composting, although not necessarily significantly (*p* > 0.05), compared to concentrations measured on day 0 (Table 2).

**Figure 4:**
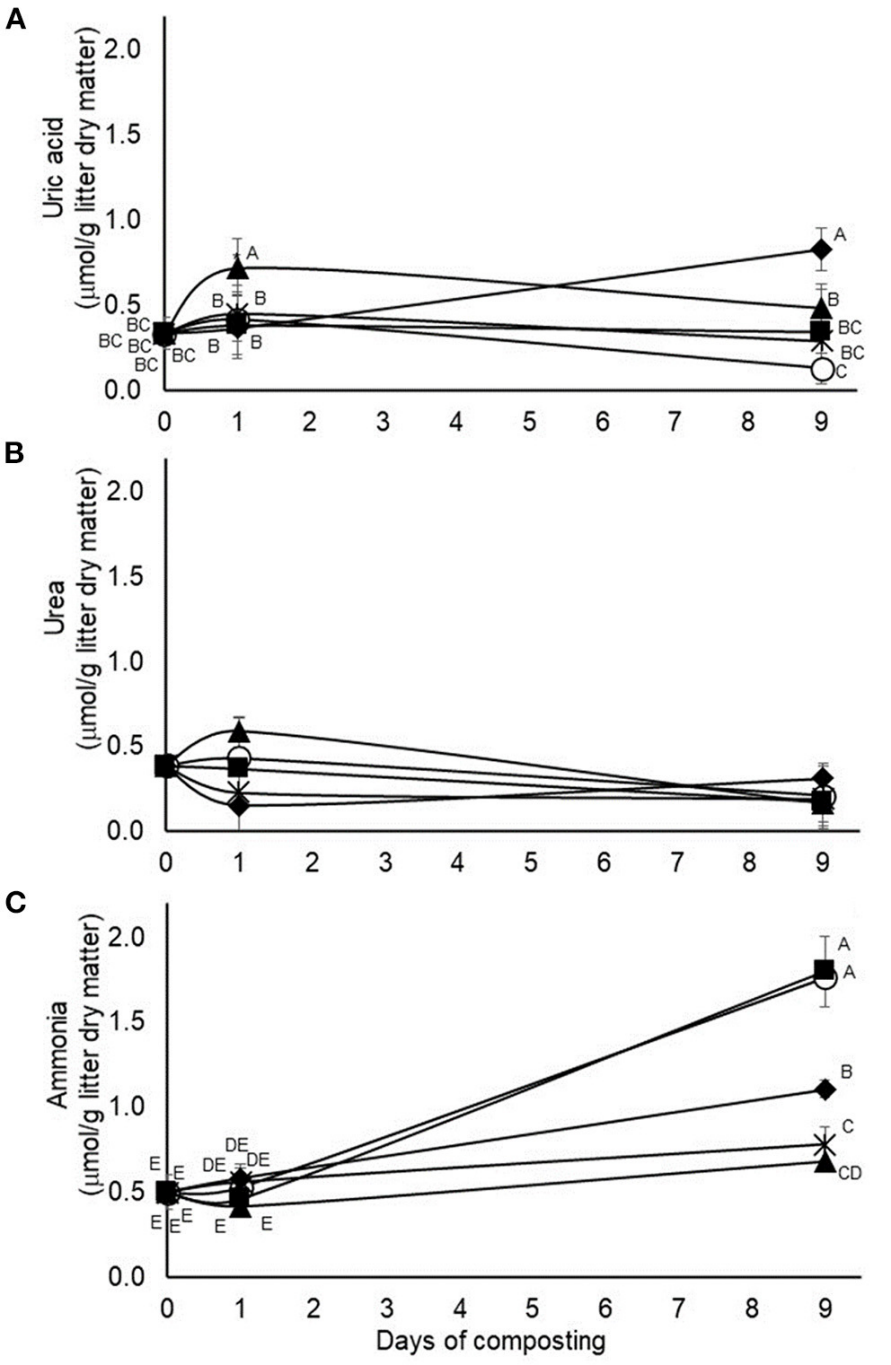
Interactions (*p* = 0.0007, 02211, and <0.0001, respectively) between treatment and time of composting on concentrations of uric acid **(A)**, urea, **(B)** and ammonia **(C)** during 9-days simulated composting without (open circles) or with 4.5% pine bark (closed squares) or 9% quebracho (closed triangles), chestnut (closed diamonds), or mimosa (X) tannins (Experiment 2). Means with unlike uppercase letters differ based on an LSD all pairwise comparison test at *p* < 0.05. Error bars denote standard deviations from *n* = 3 independent replicates at each time point.

In the second experiment, testing application of 4.5% pine bark tannin and 9% quebracho, chestnut, or mimosa tannins, treatment by time of composting interactions were also observed on accumulations of uric acid and ammonia but not on accumulations of urea (Figure 4). In the case of uric acid, accumulations were higher in quebracho-treated composts after 24 h of composting than in the other tannin-treated or untreated composts (Figure 4). After 9 days of composting, uric acid concentrations were highest (*p* < 0.05) in chestnut-treated composts, lowest (*p* < 0.05) in the untreated composts, and intermediate in the other tannin-treated composts (Figure 4). Ammonia accumulations increased during the 9-day composting period, but much more for untreated and pine bark tannin-treated composts than for composts treated with chestnut, mimosa, or quebracho tannins, with accumulations of ammonia decreasing in descending order, respectively. The main effect of treatment was not observed on urea accumulations but the main effect of time of composting was observed (*p* = 0.0205, standard error of the mean = 0.0422), with concentrations being lower after 9 days of composting than on day 1 or day 0 (0.21, 0.36 and 0.38 μmol/g litter dry matter, respectively).

## Discussion

Composting poultry litter has long been recognized as an effective biocontrol strategy to decrease the number of unwanted pathogens and antimicrobial-resistant microbes in poultry litter (19, 20). Results from our first simulated composting experiment support this concept as evidenced by the near complete elimination of populations of our challenge *S*. typhimurium and wildtype *E. coli* by 9 days of composting. However, results from our second simulated composting experiment revealed that composting may not always be an effective biocontrol strategy as the microbial populations in the litter of the second study were much more resilient and persisted at >3.5 log_10_ CFU/g of litter dry matter throughout 9 days of composting. Potential reasons for the unsuccessful compost in the second experiment are not clear but likely deal with differences between the two batches of litter. For instance, the poultry litter in the second experiment had been used to rear only two broiler flocks whereas the litter of the first experiment had reared seven flocks. Moreover, the litter in the second experiment provided higher initial populations of *E. coli* and had been inoculated to achieve nearly 2 log_10_ CFU more *S*. typhimurium/g of litter dry matter than in the first experiment. The litter of the second experiment was also considerably drier at the time of initial collection at the poultry farm than the litter used in the first experiment (98 vs. 76% dry matter, respectively) which may have subtly, as reflected in DGGE similarity profiles, and differentially affected the microbial characteristics within the two litter sources.

While tannins have been reported to exert antimicrobial activities against *S*. typhimurium, *E. coli*, and other microbes when applied in a number of different applications (11, 21–24), results from the first simulated composting experiment revealed that treatment of litter with 0.63% tannin achieved only a marginal improvement in antimicrobial activity. For instance, the 0.63% tannin treatments achieved less than a 1 log_10_ increase in anti-*S*. typhimurium activity in composts treated with quebracho tannin. Effects of tannin treatment were not observed against *E. coli* or against total culturable aerobes in the first simulated compost experiment. Treatment of poultry litter with 4.5% pine bark or 9% quebracho, chestnut, or mimosa tannins in the second compost experiment achieved no improvement in antimicrobial activity, and some of the treated composts actually enriched the microbial populations during composting. It is possible that the higher tannin administrations may have selectively inhibited some of the bacteria within the litter thereby allowing the growth of tannin-tolerant microbes (25). Mechanistically, hydrolyzable tannins such as tannic acid have been demonstrated to inhibit bacterial growth by binding iron thereby reducing its bioavailability (26, 27). Condensed tannins on the other hand are believed to be inhibitory because they can disrupt the integrity of bacterial cell walls or may bind to proteins, enzymes, or amino acids important for cellular activity (26). It is known that some microbes exhibit tolerance or resistance to tannins via a variety of degradation mechanisms and that some anaerobes may use certain tannin constituents or their degradation products as carbon or energy sources (25, 28). It is also possible that oxygen consumption within the second simulated compost experiment may have been sufficiently rapid to achieve anaerobic conditions. Anaerobic conditions would be expected to minimize the inhibitory activity of the tannins against *E. coli*, and possibly *S*. typhimurium, as it has been reported that condensed tannins inhibit *E. coli* only in the presence of oxygen (29).

Contrary to that observed by Arzola-Alvarez et al. (11), beneficial effects of the tannin treatments on uric acid accumulation were not observed in the first simulated compost experiment. There was a tendency, however, for greater retention of urea in the compost treated with 0.63% chestnut tannin when compared to untreated compost and in the composts treated with pine bark, quebracho, or mimosa tannins. There was also a tendency for an increased accumulation of ammonia in the chestnut tannin-treated compost when compared to the other tannin-treated or untreated composts. Conversely, beneficial effects of tannin treatment were observed on nitrogen retention in the second simulated compost experiment, with uric acid accumulations being increased by 42 to 84% in composted poultry litter treated with 9% quebracho- and chestnut compared to accumulations in untreated controls. Ammonia accumulations were 37 to 61% lower after 9 days of composting litter treated with 9% quebracho, chestnut, or mimosa tannins compared to accumulations in untreated compost or compost treated with 4.5% pine bark tannin. Increased uric acid concentrations in the poultry litter would yield a valuable source of crude protein when used as a ruminant feedstuff. Uric acid concentrations in the poultry litter used in the two experiments of this study ranged from 0.33 (experiment 2) to 1.98 (experiment 1) μmol/g of litter dry matter (the equivalent of 56 to 332 mg/kg of litter dry matter, respectively) were considerably lower than reported by Mowrer et al. (30) but were within the range reported earlier (31). Variability in nitrogen concentrations in different litter sources is not unexpected, however, as numerous compositional, environmental, or managerial factors such as types of bedding sources used, ventilation, number of flocks reared, moisture, and humidity as well as temperature can affect nitrogen status (32). As reported earlier (8, 30, 33), uric acid increased in concentration as early as 3 days after the beginning of composting in the present experiments thereby adding support to the conclusion that the nitrogen in uric acid may exist in a reversible flux between the end products of uric acid degradation and uric acid synthesis as proposed by Mowrer et al. (33). It seems reasonable to suspect that xanthine oxidase activity may catalyze the transformation of available purines to uric acid during the early, aerobic period of composting; however, it has been reported that xanthine oxidase activity may be inhibited by tannins (26, 34, 35). Oxidation of uric acid to allantoin by uricase activity is also thermodynamically favorable during aerobiosis which, contrary to what was observed in the present study, would thus be expected to decrease uric acid concentrations in the simulated composts (34, 36). Conversely, xanthine dehydrogenase is an anaerobic enzyme that may catalyze the conversion of xanthine to uric acid (34), however, this activity has been demonstrated in the termite gut it has received little attention in poultry litter composts (37). It seems possible that oxygen consumption by facultative anaerobes during the early period of composting may be sufficiently rapid to establish anaerobic conditions able to sustain xanthine dehydrogenase activity. Numerous facultative and strictly anaerobic bacteria are known to metabolize uric acid, some of which use a formate dehydrogenase-dependent pathway to generate electrons via the oxidation of formate to reduce nicotinamide adenine dinucleotide (34, 38–41). The dramatic increase in uric acid production in simulated composts treated with short chain nitrocompounds that inhibit formate dehydrogenase/formate hydrogen lyase activity suggests these inhibitors may inhibit the growth of uric acid-degrading bacteria (8, 42, 43) by disrupting redox homeostasis within the microbes. For instance, decreased growth of anaerobically-grown *E. coli* and *Salmonella* by nitrocompound-caused inhibition of formate dehydrogenase/formate lysase-linked hydrogen evolving hydrogenase activity has been reported (44). The decreased growth is reasoned to be due to the disruption of a major pathway for some microbes to dispose of electrons produced during fermentative processes such as glycolysis which subsequently causes feedback inhibition of energy-conserving reactions (45). Under conditions of excessive nucleotide accumulation, however, the microbial populations may compensate by redirecting electron flow away from inoperative electron sinks, such as nitrocompound-caused inhibition of hydrogen production, to dispose of electrons via reduction of other potential electron acceptors such as xanthine to yield uric acid and carbon dioxide. To our knowledge, this process has not been extensively studied in composted poultry litter.

## Conclusions

Results from our simulated compost experiments provide evidence that composting may usually but not always be effective biotechnological process to eliminate certain pathogens within poultry litter and thus there may be instances where composting may be insufficient. Results from our experiments further provide evidence that while treatment of poultry litter with certain tannin compounds had little if any beneficial antimicrobial effect, the treatments did help preserve the crude protein value of the composted poultry litter for use as a ruminant feedstuff or fertilizer. Further studies are warranted to optimize treatment formulations and applications to achieve optimal nitrogen retention.

## Data Availability Statement

The original contributions presented in the study are included in the article/supplementary material, further inquiries can be directed to the corresponding author/s.

## Author Contributions

CA-A, RA, and JS-C contributed equally to the design and planning of the studies. CA-A, RA, MH, EL, OR-B, YC-C, AA-R, MO-M, BM, LW, RC, and JS-C contributed to the conduct of the study, data analysis, interpretation of results and writing of the paper. All authors contributed to the article and approved the submitted version.

## Funding

This project was funded by research funds appropriated by the United States Department of Agriculture.

## Conflict of Interest

The authors declare that the research was conducted in the absence of any commercial or financial relationships that could be construed as a potential conflict of interest.

## Publisher's Note

All claims expressed in this article are solely those of the authors and do not necessarily represent those of their affiliated organizations, or those of the publisher, the editors and the reviewers. Any product that may be evaluated in this article, or claim that may be made by its manufacturer, is not guaranteed or endorsed by the publisher.
